# Inverse expression of hyaluronidase 2 and hyaluronan synthases 1–3 is associated with reduced hyaluronan content in malignant cutaneous melanoma

**DOI:** 10.1186/1471-2407-13-181

**Published:** 2013-04-05

**Authors:** Siiskonen Hanna, Poukka Mari, Tyynelä-Korhonen Kristiina, Sironen Reijo, Pasonen-Seppänen Sanna

**Affiliations:** 1 Institute of Biomedicine/Anatomy, University of Eastern Finland, P.O.B. 1627, Kuopio, FIN-70211, Finland; 2 Cancer Center, Kuopio University Hospital, Kuopio, Finland; 3 Institute of Clinical Medicine/Clinical Pathology, University of Eastern Finland, Kuopio, Finland; 4 Department of Clinical Pathology, Kuopio University Hospital, Kuopio, Finland; 5 Cancer Center of Eastern Finland, University of Eastern Finland, Kuopio, Finland

**Keywords:** Hyaluronan, Hyaluronan synthase, Hyaluronidase, Cutaneous tumor, Benign nevus, Melanoma

## Abstract

**Background:**

Hyaluronan is an extracellular matrix glycosaminoglycan involved in invasion, proliferation and metastasis of various types of carcinomas. In many cancers, aberrant hyaluronan expression implicates disease progression and metastatic potential. Melanoma is an aggressive skin cancer. The role of hyaluronan in melanoma progression including benign nevi and lymph node metastases has not been investigated earlier, nor the details of its synthesis and degradation.

**Methods:**

The melanocytic and dysplastic nevi, *in situ* melanomas, superficially and deeply invasive melanomas and their lymph node metastases were analysed immunohistochemically for the amount of hyaluronan, its cell surface receptor CD44, hyaluronan synthases 1–3 and hyaluronidases 1–2.

**Results:**

Hyaluronan content of tumoral cells in deeply invasive melanomas and metastatic lesions was clearly reduced compared to superficial melanomas or benign lesions. Furthermore, hyaluronan content in the stromal cells of benign nevi was higher than in the premalignant or malignant tumors. The immunopositivity of hyaluronidase 2 was significantly increased in the premalignant and malignant lesions indicating its specific role in the degradation of hyaluronan during tumor progression. Similarly, the expression of hyaluronan synthases 1–2 and CD44 receptor was decreased in the metastases compared to the primary melanomas.

**Conclusions:**

These findings suggest that the reciprocal relationship between the degrading and synthesizing enzymes account for the alterations in hyaluronan content during the growth of melanoma. These results provide new information about hyaluronan metabolism in benign, premalignant and malignant melanocytic tumors of the skin.

## Background

Malignant melanoma is an aggressive skin cancer with rapidly increasing incidence worldwide [1,2]. At the early phase, the disease can often be cured surgically, but the prognosis is worse in advanced stages resulting from its therapy resistance [3]. Although exposure to UV radiation is considered as the major risk factor for melanoma [4], a large number of nevi and atypical dysplastic nevi are associated with increased risk [5].

The dynamic extracellular matrix has been shown to contribute to cancer progression. In the skin, an abundant extracellular matrix molecule is hyaluronan (HA), which is composed of repeating disaccharide units of N-acetylglucosamine and glucuronic acid. This simple sugar molecule has been shown to enhance tumor cell invasion, proliferation and metastasis, and to promote drug resistance leading to a poor clinical prognosis (reviewed in [6]). In many malignancies of epithelial origin, *i.e.* carcinomas, the amount of hyaluronan differs from that of the normal tissue depending on the cell type. Thus, in adenocarcinomas of the breast, ovary, colon and ventricle (reviewed in [7]), the increased amount of hyaluronan correlates directly with tumor grade and poor prognosis. On the contrary, in squamous cell carcinomas (SCC) of the skin [8], mouth [9], larynx [10] and lung [11], hyaluronan content is decreased in high-grade tumors and, *e.g.*, in oral SCC is associated with poor prognosis [9]. Ultraviolet radiation, the most important risk factor for melanoma, has been shown to cause accumulation of hyaluronan and development of hyperplasia, dysplasia and SCC in mouse skin following long-term exposure, suggesting a role for hyaluronan in the early phases of malignant transformation in ultraviolet-exposed skin [12].

In a mouse model of melanoma, the amount of hyaluronan has been shown to be increased during the early stages of invasion and was localized at the interface between tumor cells and their stroma [13]. *In vitro* studies have shown that increased production of hyaluronan correlates with increased motility of melanoma cells [14] and prevention of hyaluronan synthesis by 4-methylumbelliferone (4-MU) decreases melanoma cell migration, adhesion [15,16] and invasion in 3D-melanoma cultures [17]. Melanoma cells also secrete several soluble factors like PDGF and IL-1β, which activate fibroblast hyaluronan synthesis and thus modulate the composition of the tumor stroma more favourable for cancer cell invasion and growth [18,19]. The role of hyaluronan synthases (HAS) and hyaluronan-degrading hyaluronidases (HYAL) has been investigated in many adenocarcinomas [20-23], but not widely in cancers with reduced hyaluronan expression.

In many epithelial cancers, hyaluronan content correlates positively with CD44, the main hyaluronan receptor [10,11,24]. Expression of CD44 is decreased in melanomas inversely correlating with increasing size, depth and level of invasion, while uniform expression is found in benign nevomelanocytic lesions [25]. Similarly, the levels of hyaluronan and its receptor CD44 are reduced in clinical stage I melanomas associating with poor patient prognosis [24]. However, the role of hyaluronan or details of its synthesis and degradation in other stages of the disease including benign nevi have not been investigated earlier in human tissues. In this study, we analyzed the content of hyaluronan and expression of enzymes involved in its metabolism in the human cutaneous melanocytic lesions including benign nevi, premalignant lesions, malignant melanoma and its lymph node metastases.

## Methods

### Histological specimens

This retrospective study consists of 130 specimens taken during the years 2000–2008 in Kuopio University Hospital. The study was approved by the Ethics committee of the Kuopio University Hospital and by The Finnish National Supervisory Authority for Welfare and Health. The samples consisted of 29 benign nevi (14 intradermal, 10 compound and 5 junctional nevi), 28 dysplastic nevi, 18 *in situ* melanomas, 17 superficial melanomas (invasion depth < 1 mm), 19 deep melanomas (invasion depth > 4 mm) and 19 lymph node (LN) metastases. After biopsy, the tissue samples were fixed in 10% buffered formaldehyde, embedded in paraffin and sectioned 5 μm thick. For evaluation of staining coverage and intensity, the tissue sections were stained for hyaluronan, hyaluronan receptor CD44, hyaluronan synthases 1–3 (HAS1-3) and hyaluronan degrading hyaluronidases 1–2 (HYAL1-2) as described below.

### Hyaluronan staining

The sections were rehydrated in descending xylene-ethanol series, and incubated first with 3% H_2_O_2_ for 5 min to block endogenous peroxidases, and then with 1% bovine serum albumin (BSA) in 0.1 M Na-phosphate buffer, pH 7.0 (PB) for 30 min at 37°C to block unspecific binding of the probe, followed by overnight incubation at 4°C with 2 μg/ml biotinylated hyaluronan binding complex (bHABC), isolated from bovine articular cartilage and containing the biotinylated complex of link protein and G1 domain of aggrecan [26]. After washes with PB, the sections were incubated with avidin-biotin peroxidase (1:200, Vector Laboratories, Irvine, CA) for 1 h. The color was developed with 0.05% 3,3^′^-diaminobenzidine (DAB, Sigma, St.Louis, MO) containing 0.03% H_2_O_2_. The sections were counterstained with Mayer’s hematoxylin for 2 min, washed, dehydrated, and mounted in DePex (BDH Laboratory Supplies, Poole, England). The specificity of the staining was controlled by predigesting the sections with *Streptomyces* hyaluronidase (100 TRU/ml in acetate buffer, pH 5.0 for 3 h at 37°C; Seikagaku, Kogyo, Tokyo, Japan) in the presence of protease inhibitors (Additional file 1: Figure S1).

### CD44 staining

Specimens were rehydrated as described above. After blocking the endogenous peroxidase activity in 1% H_2_O_2_ for 5 min and unspecific binding as described above, the sections were incubated overnight at 4°C with the primary antibody Hermes3 (a kind gift from Dr. Sirpa Jalkanen, University of Turku, Finland) diluted in 1:200 in 1% BSA-PB. The sections were sequentially incubated with a biotinylated anti-mouse secondary antibody (1:150, Vector Laboratories, Burlingame, CA, USA) for 1 h at room temperature. Avidin-biotin peroxidase and DAB treatments as well as the counterstaining with Mayer’s hematoxylin were carried out as described above. Hermes 3 detects an epitope in the standard backbone of CD44 and therefore also all splice variants of CD44. Control sections were stained similarly, but omitting the primary antibody.

### HAS and HYAL stainings

The deparaffinized sections were incubated in 10 mM citrate buffer, pH 6.0 for 15 min in a pressure cooker at 120°C, washed with PB, and treated for 5 min with 1% H_2_O_2_ to block endogenous peroxidase activity. Thereafter the sections were incubated in 1% BSA, 0.05% Tween-20 and 0.1% gelatine (Sigma G-2500, Sigma) in PB for 30 min to block nonspecific binding. The sections were incubated overnight at 4°C with polyclonal antibodies diluted in 1% BSA for HASes (Santa Cruz Biotechnology, Santa Cruz, CA: sc-34021 for HAS1 in 1:100, sc-34067 for HAS2 in 1:120 and sc-34204 for HAS3 in 1:80) or with the primary antibodies for HYALs (HPA002112 from Atlas Antibodies, Stockholm, Sweden for HYAL1 in 1:100 and Ab68608 from Abcam, Cambridge, UK for HYAL2 in 1:100) followed by 1 h incubation with biotinylated anti-goat antibody (1:1000, Vector Laboratories) for HASes or with biotinylated anti-rabbit antibody (1:200, Vector Laboratories) for HYALs. Visualization of the bound antibodies, counterstaining and mounting in DePex were carried out as described above. Control sections were stained similarly, but omitting the primary antibody (Additional file 1: Figure S1). In addition, the specificity of the HAS antibodies was tested with corresponding peptides as described in [12] (Additional file 1: Figure S1).

### Evaluation of stainings

The stained sections were evaluated for staining coverage and intensity separately in melanocytic cells and in the stroma surrounding the lesion by two independent observers (HS, MP). The area of the staining was estimated with a five-level scoring system from 0 to 4. Score 0 = negative was given when less than 5% of the cells were positive. Score 1 was given when 6-25% of the cells were positive, score 2 when 26-50% of the cells were positive, score 3 when 51-75% of the cells were positive and score 4 when 76-100% of the cells were positive. The intensity of the staining was estimated with a four-level scale from 0 to 3 as negative (0), weak (1), moderate (2) or strong (3) (Additional file 2: Figure S2).

### Statistical analysis

IBM SPSS Statistics 20 (IBM Corporation, Armonk, New York, USA) was used for the statistical analysis of the data. The different histological groups were compared with Mann–Whitney U-test for statistically significant differences between the groups. A difference was considered statistically significant when the p-value was less than 0.05. Correlations between the groups were tested with Spearman’s rho. A correlation coefficient stronger than 0.5 with a p-value less than 0.05 was considered significant.

## Results

### Hyaluronan content is reduced in the malignant melanocytic lesions

**Figure 1:**
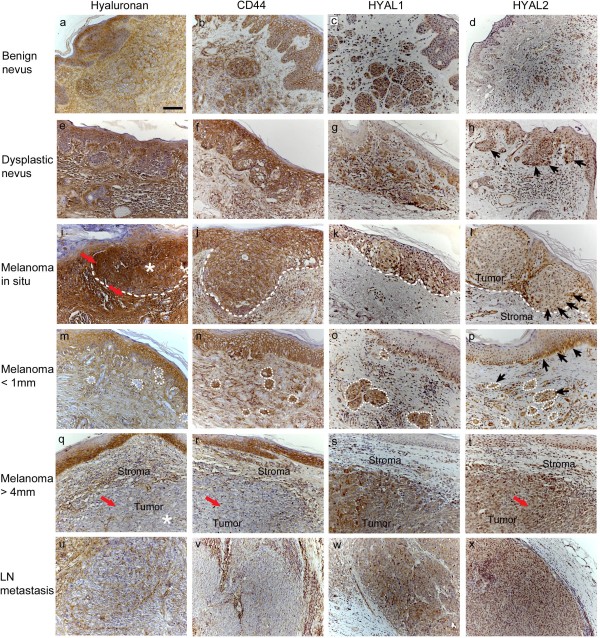
**Hyaluronan, CD44 and hyaluronidase (HYAL) 1–2 stainings of benign (a-d) and dysplastic nevi (e-h), *in situ *melanomas (i-l), superficial (<1 mm, in m-p) and deep melanomas (>4 mm, in q-t) and lymph node (LN) metastases (u-x).** Red arrows in (**i**) indicate the increased hyaluronan staining intensity of melanocytic cells and stroma of *in situ* melanoma, showing also intracellular staining of hyaluronan. Red arrow in (**q**) points to reduced hyaluronan staining and in (**r**) to reduced CD44 staining, while in (**t**) the arrow indicates the increased HYAL2 staining in deep melanomas. Black arrows in (**h**, **l**, **p**) indicate the increased HYAL2 staining in the melanocytic cells of dysplastic nevus (**h**), *in situ* melanoma (**l**) and superficial melanoma (**p**). White asterisk in (**i**) indicates strong hyaluronan staining in tumor cells in *in situ* melanoma, while in (**q**) the white asterisk shows the reduced hyaluronan staining of tumor cells in deep melanoma. White dash lines in (**i**-**p**) delineate the border between the tumor cells and the stroma in *in situ* melanomas (**i**-**l**) and encircle the invasive tumor cells in superficial melanomas (**m**-**p**). Scale bar in (**a**) 100 μm.

Hyaluronan staining was mainly localized in the pericellular matrix of the melanocytic cells, although intensive diffuse intracellular staining was also observed in almost all samples (Figure 1). Compared to benign nevi, hyaluronan appeared to be widely present also in dysplastic nevi and melanomas, but its intensity and thus the content of hyaluronan in the tissue became first increased in *in situ* melanomas and then later clearly decreased in invasive melanomas. The staining pattern of LN metastases was similar to deep melanomas.

**Figure 2:**
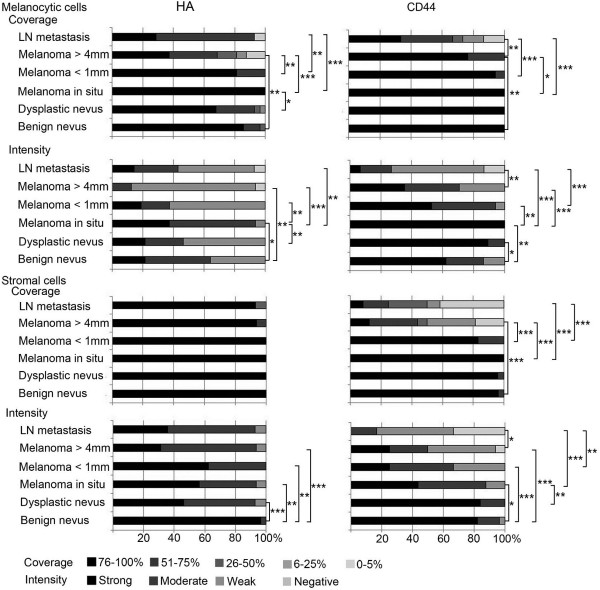
**Intensity and coverage of hyaluronan (HA) and CD44 stainings in benign and dysplastic nevi, *in situ *melanomas, superficial (<1 mm) and deep (>4 mm) melanomas and lymph node (LN) metastases.** Statistical differences between the groups are indicated by brackets. The statistical significance of the differences was tested with Mann–Whitney U-test. * p <0.05, ** p <0.01 and *** p <0.001.

In all lesions studied most melanocytic cells were hyaluronan positive (Figures 1 and 2). Interestingly, the proportion of hyaluronan positive cells was highest in *in situ* melanomas (76-100%) and the lowest in deep melanomas (51-75%) and lymph node (LN) metastases (51-75%). The percentage of positive hyaluronan staining turned out to be significantly reduced in deep melanomas compared to benign nevi (p=0.001), in situ melanomas (p=0.000) and also superficial melanomas (p=0.007). In benign nevi, the intensity of hyaluronan staining in melanocytic cells was moderate in average. Similar to the coverage, also the intensity of hyaluronan staining in melanocytic cells was highest in *in situ* melanomas (mainly moderate or strong) and lowest in deep melanomas (mainly weak) (Figures 1 and 2), these changes being statistically significant compared to benign nevi (p=0.048 and p=0.001, respectively). Intensity of hyaluronan staining in superficial melanomas varied from weak to strong and was similar to benign nevi.

Hyaluronan positive area covered most of the stromal tissue (76-100% in average) in all groups without any significant differences among the groups. The staining intensity of the stromal tissue was strong in benign nevi, whereas other lesions displayed moderate to strong staining with few samples with weak intensity. The reduction of stromal hyaluronan staining intensity in the other lesion types was statistically significant (p=0.003-0.000).

### The staining of CD44 is reduced in tumor and stromal cells of malignant melanocytic lesions

Immunostaining of the hyaluronan receptor CD44 showed relatively similar staining pattern as hyaluronan in different stages of melanoma. CD44 localized on the plasma membrane of melanocytic cells, making often a reticular pattern in the tissue (Figure 1). Like hyaluronan, CD44 was strongly expressed in all lesions studied (Figures 1 and 2), more than 76% of the melanocytic cells being positively stained in all benign nevi, dysplastic nevi and *in situ* melanomas and in almost all superficial melanomas (Figure 2). About one fourth of deep melanomas presented reduced staining coverage (51-75%) (Figure 2), differing significantly from benign nevi (p=0.007). The proportion of CD44-positive melanocytic cells was even lower (in 67% of samples coverage less than 76%) in LN metastases compared to deep melanomas (p=0.006).

The CD44 staining intensity in melanocytic cells was mostly either moderate or strong in other lesions except LN metastases which showed weaker staining (Figures 1 and 2). CD44 staining intensity displayed similar pattern of increased staining in premalignant lesions as hyaluronan, being increased in dysplastic nevi and *in situ* melanomas (p=0.013 and p=0.004, respectively) compared to benign nevi. The moderate intensity of CD44 staining found in superficial and deep melanomas was thus decreased compared to strong staining in *in situ* melanomas (p=0.002 and p=0.000, respectively). Similar to the coverage of CD44 staining, also the intensity of CD44 staining was further decreased in LN metastases compared to deep melanomas (p=0.007).

In stroma, the coverage of CD44 staining was high (76-100% in average) in benign and dysplastic nevi, *in situ* melanomas and superficial melanomas and was not statistically different among these groups. Interestingly, the stromal staining coverage was 26-50% in average in deep melanomas, significantly decreased from the level found in benign nevi (p=0.000), *in situ* melanomas (p=0.000) and superficial melanomas (p=0.000). The stromal coverage of CD44 was only 6-25% in average in LN metastases, but it was not significantly different than in deep melanomas. The intensity of stromal CD44 staining was strong in benign and dysplastic nevi, all other lesions showing reduced staining intensity compared to benign nevi (p=0.010-0.000). The intensity of CD44 staining was mostly weak in LN metastases, significantly reduced from the level in deep melanomas (p=0.020).

### The expression of HYAL2 is increased in melanocytic lesions

**Figure 3:**
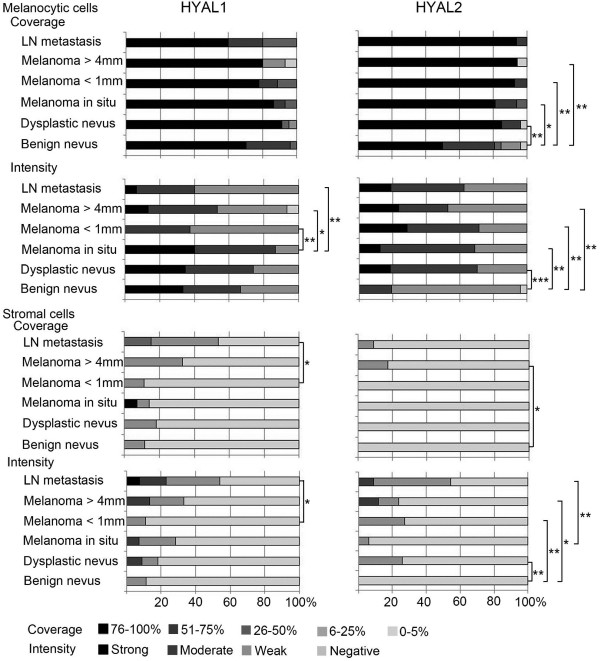
**Intensity and coverage of hyaluronidase (HYAL) 1–2 stainings in benign and dysplastic nevi, *in situ *melanomas, superficial (<1 mm) and deep (>4 mm) melanomas and lymph node (LN) metastases.** Statistical differences between the groups are indicated by brackets. The statistical significance of the differences was tested with Mann–Whitney U-test. * p <0.05, ** p <0.01 and *** p <0.001.

Especially the staining of HYAL2 was changed in dysplastic nevi and melanomas compared to benign nevi, providing a logical explanation for the observed alterations in hyaluronan staining, but also suggesting a connection to the reduced staining of CD44 as previously reported [27]. Cells showing HYAL1 or HYAL2 positive immunostaining were mainly melanocytic cells, while the majority of stromal cells were negative (Figure 1). Hyaluronidase positive staining was localized intracellularly, spreading diffusely throughout the cytoplasm (Figures 1 and 3).

In all groups, the proportion of HYAL1-positive melanocytic cells was high (76-100%), however, the staining intensity was mainly either weak or moderate without any significant differences compared to benign nevi (Figure 3). Interestingly, the intensity of HYAL1 in melanocytic cells was significantly reduced in superficial (p=0.008) and deep melanomas (p=0.029) and also in LN metastases (p=0.005) compared to *in situ* melanomas.

The proportion of HYAL2 positive melanocytic cells varied from 0-5% to 76-100% in benign nevi, and was either weak or moderate when present, while in other melanocytic lesions over 76% of cells were positive and the staining intensity was higher, varying from weak to moderate or even strong (Figures 1 and 3). Differences between benign nevi and the other lesions studied in the HYAL2 staining in melanocytic cells were statistically significant (p=0.042-0.006 for coverage and p=0.009-0.000 for intensity). The HYAL2 staining in melanocytic cells was similar in LN metastases as in melanomas.

HYAL2 immunostaining in the stromal cells was very modest in all groups studied, just a few cells were positively stained and the intensity of the positive staining when detected was weak (Figure 3). However, higher proportion of stromal cells were HYAL2 positive (6-25%) in deep melanomas (p=0.028), and the intensity of stromal HYAL2 staining was higher in dysplastic nevi (p=0.006) and in superficial (p=0.006) and deep melanomas (p=0.010) compared to benign nevi. In the stroma, the intensity of HYAL2 immunoreactivity correlated negatively with hyaluronan staining intensity in superficial melanomas (correlation coefficient -0.655, p=0.040).

### The expression of Hyaluronan synthase 1 and 2 is decreased in melanomas

**Figure 4:**
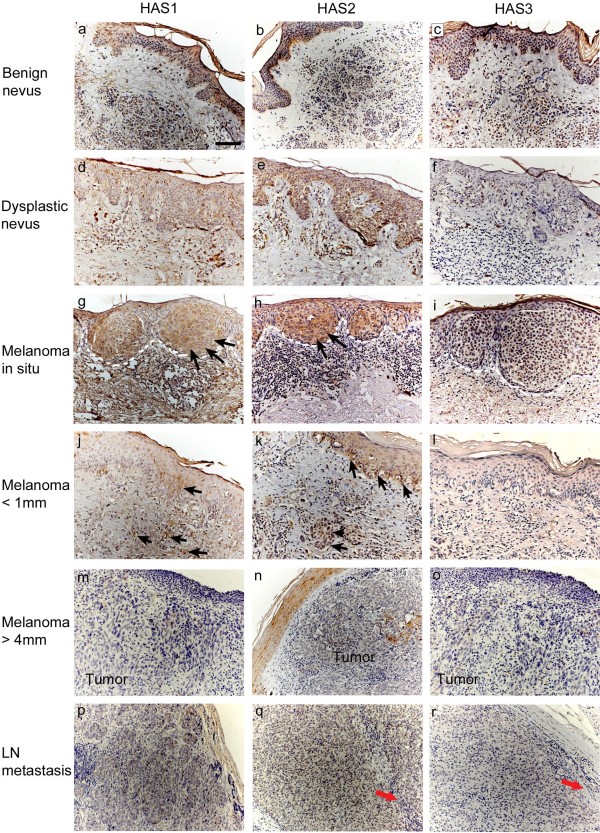
**Hyaluronan synthase (HAS) 1–3 expression in benign (a-c) and dysplastic (d-f) nevi, *in situ *melanomas (g-i), superficial (j-l) and deep melanomas (m-o) and lymph node (LN) metastases (p-r).** Black arrows in (**g**-**h**) and (**j**-**k**) point to tumor cells in *in situ* melanomas (**g**-**h**) and superficial melanomas (**j**-**k**) stained positively for HAS1 (**g**, **j**) and HAS2 (**h**, **k**). White dash lines in (**g**-**i**) delineate the border between the tumor cells and the stroma in *in situ* melanomas. Red arrow in (**q**) and in (**r**) point to stroma of LN metastases negative for HAS2 and HAS3, respectively. Scale bar in (**a**) 100 μm.

**Figure 5:**
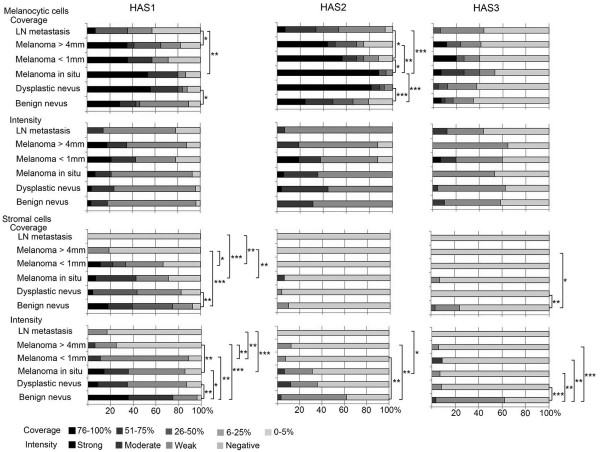
**Intensity and coverage of hyaluronan synthase (HAS) 1–3 stainings in benign and dysplastic nevi, *in situ *melanomas, superficial (<1 mm) and deep (>4 mm) melanomas and lymph node (LN) metastases.** Statistical differences between the groups are indicated by brackets. The statistical significance of the differences was tested with Mann–Whitney U-test. * p <0.05, ** p <0.01 and *** p <0.001.

Immunostaining with HAS1-3 specific antibodies showed positive staining in all samples studied regardless of the lesion type (Figures 4 and 5). However, the proportion of positive cells in the melanocytic and stromal cells and subcellular localization of the staining showed differences among the HAS isoforms. While most melanocytic cells showed positive immunostaining for HAS1 and HAS2, less than half of them were positive for HAS3. In stromal cells HAS1 appeared to be the prevalent isoform, while HAS2 and HAS3 were less abundant (Figures 4 and 5). Immunostaining for HAS1-3 proteins was detected homogenously in the cytoplasm and at the plasma membrane.

The proportion of HAS1 positive melanocytic cells tended to be higher in dysplastic nevi and melanomas compared to benign nevi, although the difference was statistically significant only for dysplastic nevi (p=0.021). In LN metastases the proportion of HAS1 positive melanocytic cells was lower (6-25% in average) than in deep melanomas (26-50% in average, p=0.039). Despite the variation in staining coverage, the staining intensity for HAS1 in melanocytic cells showed no differences between the lesion groups.

In benign nevi, in average 26-50% of the stromal cells were HAS1 positive, while in dysplastic nevi the proportion of HAS1 positive cells was lower (6-25% in average, p=0.005). *In situ* melanomas and superficial melanomas resembled benign nevi, while in deep melanomas the proportion of HAS1 positive cells was lower (typically less than 6%, p=0.000) and only occasional HAS1 positive stromal cells were found in LN metastases (Figure 5).

The HAS1 staining intensity in stromal cells showed similar trend as the coverage. The highest intensities were found in benign nevi, scored mainly as moderate or strong, followed by dysplastic nevi and *in situ* melanomas showing weak to moderate staining, and superficial and deep melanomas showing mainly weak or negative staining, respectively (Figure 5). All these groups differed significantly from benign nevi (p=0.020-0.000). The stromal HAS1 staining in LN metastases was similar to deep melanomas.

Staining of the melanocytic cells for HAS2 showed similar trends as HAS1 (Figure 5). The proportion of the melanocytic cells stained positively for HAS2 was significantly higher in dysplastic nevi (mean 76-100%, p=0.000) and *in situ* melanomas (mean 76-100%, p=0.000) compared to benign nevi (mean 26-50%). In superficial and deep melanomas, the proportion of HAS2 positive melanocytic cells was significantly lower (mean 51-75% and 26-50%, respectively) than in *in situ* melanomas (p=0.043 and p=0.007, respectively).

The HAS2 staining intensity of melanocytic cells was weak in general and did not differ between the groups. Only few stromal cells were positively stained for HAS2 in all groups. The staining intensity for HAS2 in the stroma was generally low, varying from negative to moderate in benign and dysplastic nevi and *in situ* melanomas, and from weak to negative in superficial (p=0.001) and deep melanomas (p=0.002) compared to benign nevi. Interestingly, the stroma of LN metastases was negative for HAS2.

HAS3 staining was the weakest of the three hyaluronan synthases. In half of the specimens practically no staining was observed in melanocytic cells (Figure 5). The staining intensity, when detected, was generally weak, and there were no constant differences between the different lesions in HAS3 staining coverage or intensity in melanocytic cells. HAS3 positive stromal cells were detected more in benign nevi (6-25% in one fifth of the samples, although 0-5% in general), while in other groups the proportion of HAS3 positive stromal cells was very low (only 0-5%). The stromal coverage of HAS3 was significantly reduced in dysplastic nevi and deep melanomas compared to benign nevi (p=0.009 and p=0.030, respectively). The intensity of stromal HAS3 was mostly weak in benign nevi, and negative in other lesions (Figure 5, p=0.009-0.000). Interestingly, also HAS3 staining was totally absent in the stroma of LN metastases.

## Discussion

There is a growing evidence that hyaluronan has an important role in promoting tumor progression of epithelial malignancies (reviewed in [6]). In the present work we studied for the first time the expression pattern of hyaluronan-metabolizing enzymes and the hyaluronan content in different stages of cutaneous melanocytic lesions. The current study indicates that hyaluronan content is increased in the melanocytic cells during the early stages of melanoma (*in situ* melanoma phase), but is thereafter declined. The staining of CD44 was similar to the pattern of hyaluronan staining. Our results suggest that the reduction of hyaluronan in the invasive melanoma is due to increased expression of hyaluronidase 2 and decreased expression of hyaluronan synthases 1–3.

These results are in line with the findings of reduced hyaluronan level in cutaneous stage I melanoma [24]. Thus, the loss of hyaluronan receptor CD44 in melanocytic cells accompanied the decreased hyaluronan content. As CD44 has multifaceted role in the hyaluronan metabolism acting both in ligation of hyaluronan on the cell surface [28-30] and in its endocytosis [31,32], the significance of its loss for hyaluronan metabolism in melanoma is somewhat difficult to deduce. The role of other players involved in hyaluronan metabolism like synthases and hyaluronidases have not been previously studied in melanoma progression. Our data suggest that changes in the expression of both synthetic and degrading enzymes occur during melanoma progression, and they have different temporal and spatial distributions.

The amount of hyaluronan seems to be biphasic in premalignant and malignant melanocytic lesions. First, in dysplastic nevi the expression of HAS1 and HAS2 in the melanocytic cells is increased, although at this stage the amount of hyaluronan is not yet different from benign lesions. Then in *in situ* melanomas, the proportion of HAS2 positive melanocytic cells is higher than in benign nevi, and at this state the hyaluronan content is also increased in melanocytic cells. This may indicate the accumulation of hyaluronan behind the intact basement membrane before the invasive phase has been achieved. Instead, in the deep melanomas the tumor cells show markedly reduced amount of hyaluronan, which is associated with the increased expression of HYAL2. Similarly in the stromal compartment hyaluronan content is significantly decreased in melanomas compared to benign lesions, concurrent with increased activity of HYAL2 and decreased expression of hyaluronan synthases. Although melanoma originates form melanocytes and not from the stratified epidermis as such, similar tendency to increased hyaluronan staining in premalignant or early malignant lesions and decreased staining in more advanced tumors has been reported earlier in squamous cell carcinomas of oral [9], laryngeal [10], esophageal [33] and skin [8] epithelium, all originating from stratified epithelium.

It is possible that the elevated hyaluronan content in *in situ* melanomas promote the very early events of carcinogenesis by facilitating cell migration [14,34,35] and proliferation [36,37], and by protecting cancer cells from apoptosis [38]. These effects of hyaluronan involve signaling through CD44 [39-41]. Hyaluronan, CD44 and another hyaluronan receptor, receptor for hyaluronan mediated motility (RHAMM) have been shown to form signaling complexes with extracellular regulated kinase 1/2 (ERK1/2), leading to increased motility of breast cancer cells [42]. ERK1/2 is a member of the central RAS pathway activated in nearly all melanomas [43], possibly linking hyaluronan and CD44 signaling with the activation of this pathway early in melanoma. The decreased hyaluronan content found in advanced melanomas may also be important for the tumor progression, as the clearance of the extracellular water-attractive gel-like hyaluronan may give more space for inflammatory cells like mast cells [44], which produce a range of cancer-cell stimulating cytokines and chemokines [45,46] and thus further enhance the malignant phenotype of the transformed cells.

HYAL2 expression was significantly increased in all premalignant and malignant melanocytic lesions compared to the benign nevi. In the urothelial carcinoma [47], prostate adenocarcinoma [48] and breast carcinoma [49] HYAL1 has been considered the mostly expressed tumor-derived hyaluronidase. In our specimens, the staining pattern of HYAL1 was not altered in the lesions compared to benign nevi, but the intensity of HYAL1 in melanocytic cells was decreased in superficial and deep melanoma and lymph node metastases compared to *in situ* melanomas. The presence of hyaluronidase in tumor cells has been shown to increase angiogenesis *in vivo*[50]. Hyaluronan oligosaccharides produced by hyaluronidases mediate the angiogenic effects [51,52] and may also activate matrix metalloproteinases enhancing the invasion of the tumor [53]. Interestingly, in a mouse model of prostate cancer, co-expression of a hyaluronidase (HYAL1) and a hyaluronan synthase (HAS2) significantly increased angiogenesis [22]. An upregulation of both HYAL2 and HAS1-2 in dysplastic nevi and *in situ* melanomas was also observed in the present study. In our specimens, the hyaluronan binding probe used in the hyaluronan stainings detects hyaluronan oligosaccharides larger than 10 sugars, thus possibly detecting some of the fragmented hyaluronan. However, it is also possible that some smaller (<HA10) oligosaccharides still exist in the tissue, if not diluted away during sample processing.

Previously, the role of hyaluronan in metastases has not been widely studied. High amount of stromal hyaluronan in the primary tumor has been shown to associate with metastasis in prostate [54] and thyroid [55] cancer, both representing tumors from simple epithelium. In tumors from stratified epithelium, irregular and locally reduced staining of hyaluronan and CD44 in the primary tumor associated with high frequency of metastasis in laryngeal squamous cell carcinoma [10]. However, the staining pattern of the metastases was not analyzed in these studies. In a scid mouse model of human adenocarcinomas, lung metastases of colon carcinoma showed similar hyaluronan staining pattern as the primary tumor, but hyaluronan synthases were absent [56]. In our data, the staining pattern of the lymph node metastases resembled that of the primary tumor, but the amount of CD44 and HAS1 were further reduced in the metastases compared to deep melanomas. These results suggest that the decreased hyaluronan content in lymph node metastases is explained by reductions in its synthesis and binding at the cell surface.

## Conclusions

Our data show for the first time the biphasic pattern of hyaluronan metabolism in cutaneous melanocytic tumors revealing an increased hyaluronan synthesis in premalignant lesions followed by reduced hyaluronan content in malignant melanoma as a consequence of decreased HAS expression and increased amount of degradative HYAL2. Further studies are needed to clarify the prognostic power of HYAL2 upregulation and HAS1-3 downregulation in melanoma.

## Abbreviations

bHABC: Biotinylated hyaluronan binding complex; BSA: Bovine serum albumin; HA: Hyaluronan; HAS: Hyaluronan synthase; HYAL: Hyaluronidase; LN: Lymph node; PB: Phosphate buffer.

## Competing interests

The authors declare that they have no competing interests.

## Authors’ contributions

HS analyzed the specimens, put together the data, performed statistical analyses and drafted the manuscript. MP analyzed the specimens and commented on the manuscript. KTK participated in design of the study and commented on the manuscript. RS and SPS participated in design of the study, coordinated the study, and helped to draft the manuscript. All authors read and approved the final manuscript.

## Pre-publication history

The pre-publication history for this paper can be accessed here:

http://www.biomedcentral.com/1471-2407/13/181/prepub

## Supplementary Material

Additional file 1: Figure S1 Control stainings showing the specificity of the probe used for staining of hyaluronan (a-b) and antibodies used to detect HAS1-3 (c-h) and HYAL1-2 (i-l) are shown in *in situ* melanomas (a-b for HA and c-h for HAS1-3) and in superficial melanomas (invasion <1 mm) (i-l for HYAL1-2). To check the specificity of hyaluronan staining (a, white asterisk), the sections were pre-digested with 100 TRU/ml *Streptomyces* hyaluronidase in the presence of protease inhibitors removing hyaluronan from the sections (b, black asterisk). Red dash lines in (a-b) delineate the border between the tumor cells and the stroma. To confirm the specificity of the HAS stainings (c-h), the primary antibodies (HAS1 in (c), HAS2 in (e) and HAS3 in (g), white arrows in (c) and (e)) were preincubated with the corresponding peptides (peptide block of HAS1 in (d), HAS2 in (f) and HAS3 in (h), black arrows in (d) and (f)) used in the immunization. For HYAL stainings (HYAL1 in (i) and HYAL2 in (k), red asterisks), the specificity was checked by omitting the primary antibody (HYAL1 in (j) and HYAL2 in (l), red asterisks). Scale bar in (l) 100 μm. Click here for file

Additional file 2: Figure S2 Different intensity levels of HYAL2 staining in deep melanomas (a-c) shown as an example of the scoring. The intensity of the staining was estimated with a four-level scale as negative, weak (a), moderate (b) or strong (c). Scale bar in (a) 100 μm. Click here for file
