# Supplementary figures and images for: Inverse expression of hyaluronidase 2 and hyaluronan synthases 1–3 is associated with reduced hyaluronan content in malignant cutaneous melanoma
Source: BMC Cancer. 2013 Apr 5;13:181. doi: 10.1186/1471-2407-13-181 (PMC3626669; doi:10.1186/1471-2407-13-181)

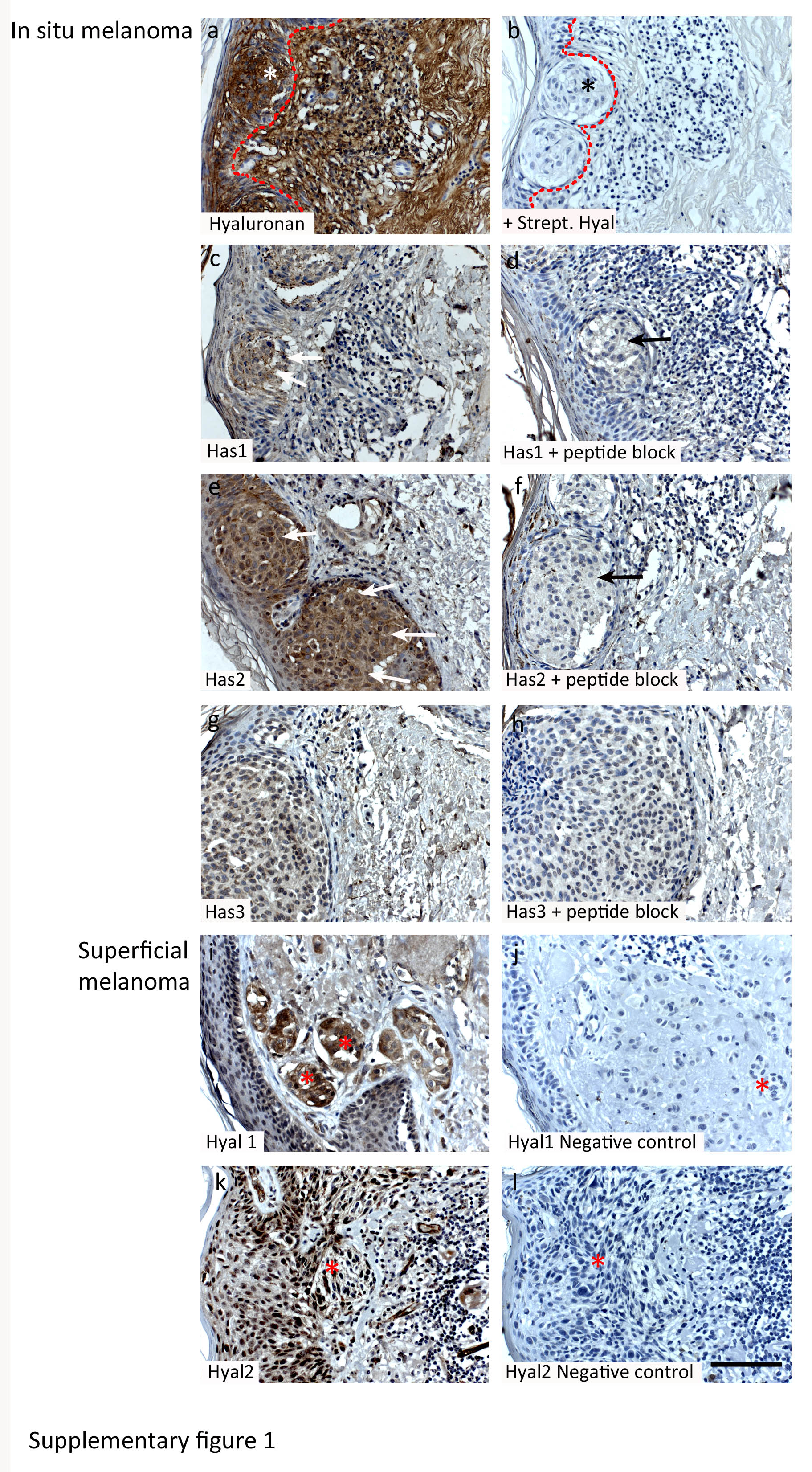

Supplement: Additional file 1: Figure S1 — Control stainings showing the specificity of the probe used for staining of hyaluronan (a-b) and antibodies used to detect HAS1-3 (c-h) and HYAL1-2 (i-l) are shown in in situ melanomas (a-b for HA and c-h for HAS1-3) and in superficial melanomas (invasion <1 mm) (i-l for HYAL1-2). To check the specificity of hyaluronan staining (a, white asterisk), the sections were pre-digested with 100 TRU/ml Streptomyces hyaluronidase in the presence of protease inhibitors removing hyaluronan from the sections (b, black asterisk). Red dash lines in (a-b) delineate the border between the tumor cells and the stroma. To confirm the specificity of the HAS stainings (c-h), the primary antibodies (HAS1 in (c), HAS2 in (e) and HAS3 in (g), white arrows in (c) and (e)) were preincubated with the corresponding peptides (peptide block of HAS1 in (d), HAS2 in (f) and HAS3 in (h), black arrows in (d) and (f)) used in the immunization. For HYAL stainings (HYAL1 in (i) and HYAL2 in (k), red asterisks), the specificity was checked by omitting the primary antibody (HYAL1 in (j) and HYAL2 in (l), red asterisks). Scale bar in (l) 100 μm. [file 1471-2407-13-181-S1.jpeg]

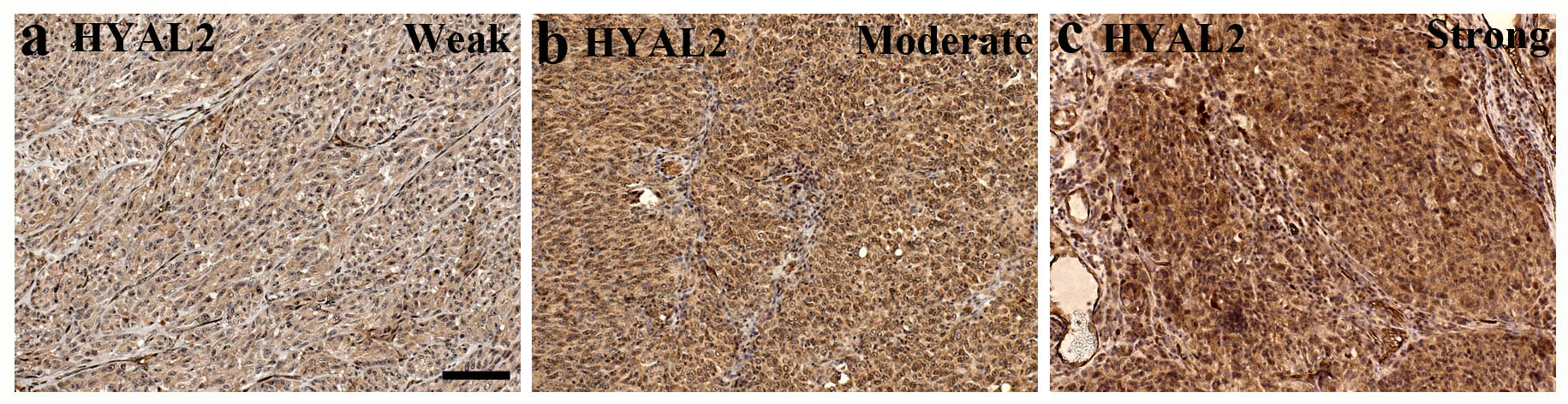

Supplement: Additional file 2: Figure S2 — Different intensity levels of HYAL2 staining in deep melanomas (a-c) shown as an example of the scoring. The intensity of the staining was estimated with a four-level scale as negative, weak (a), moderate (b) or strong (c). Scale bar in (a) 100 μm. [file 1471-2407-13-181-S2.jpeg]
